# Introductory Address

**Published:** 1853-04

**Authors:** 


*Introductory Address,* by Professor L. Reuben, *to the Medical Class of the Worcester Medical Institution.*

Professor Reuben has said some very good things in his address—and shows decidedly that he is a thorough going medical reformer as he understands it—and would make all allopathists, as he is pleased to style them, entirely antagonistic, and utterly irreconcilable with this new system of reform—eclectic reform—of which, it seems, he is an expounder and teacher. He would have us believe that this new system *is all in all.* That it is the only one that is at all perfect, as it most happily unites both the "philosophy and practice," and one which must consequently undermine and ultimately supercede every other system.

With all due respect to the professor, we must say that his address clearly shows the structure of a mind, whose elements have been educated far more in the regions of fancy than in the rigid school of facts and analysis. He says that the allopathic, or regular medical schools, have good teachers of the philosophy, principles, or doctrines of medicine, but they lack the other important half, i. e. the practice. And then, on the other hand, the physicians of this allopathic school who engage in practice, fail to unite in their practice, that other important half the philosophy. And that he and his colleagues, if we understand the spirit of his remarks, are the only fortunate few who have effected this happy combination, which is, doubtless, very speedily destined to show its superior skill, in vanquishing disease by its magic touch, and thus realize the certainty of the professor's expectations in the practice of his philosophy.

But is it not strange that Professor Reuben has never heard of hospitals, infirmaries, and clinical lectures, being associated with every regular medical school, both at home and abroad of any standing. And if so, how can he call the whole of this extensive arrangement for practice, and nothing else—only teaching the principles? We must confess an utter loss for any explanation, save in a sort of perverted imagination, which blinds the mind to every thing but its own especial and particular fancy.

